# Important Design Features of Personal Health Records to Improve Medication Adherence for Patients with Long-Term Conditions: Protocol for a Systematic Literature Review

**DOI:** 10.2196/resprot.9778

**Published:** 2018-06-28

**Authors:** Elisavet Andrikopoulou, Philip James Scott, Helena Herrera

**Affiliations:** ^1^ School of Computing Faculty of Technology University of Portmouth Portsmouth United Kingdom; ^2^ School of Pharmacy and Biomedical Sciences Faculty of Science University of Portmouth Portsmouth United Kingdom

**Keywords:** personal health records, medication adherence, chronic condition, comorbidities, drug regime, long-term conditions

## Abstract

**Background:**

The National Health Service (NHS) England spent £15.5 billion on medication in 2015. More than a third of patients affected by at least one long-term condition do not adhere to their drug regime. Many interventions have been trialed to improve medication adherence. One promising innovation is the electronic personal health record.

**Objective:**

This systematic literature review aims to identify the important design features of personal health records to improve medication adherence for patients with long-term conditions.

**Methods:**

This protocol follows the Preferred Reporting Items for Systematic Reviews and Meta-Analyses Protocol (PRISMA-P 2015) statement. The following databases will be searched for relevant articles: PubMed, Science Direct, BioMed Central, Cumulative Index to Nursing and Allied Health Literature, Cochrane Database of Systematic Reviews, and the Cochrane Central Register of Controlled Trials. Studies published in the last fifteen years, in English, will be included if the participants are adults who were treated outside the hospital, have the ability to self-administer their medication, and have at least one long-term condition. The review will exclude commercial or political sources and papers without references. Papers that research pediatrics, pregnant, or terminally ill patients will also be excluded, since their medication management is typically more complex.

**Results:**

One reviewer will screen the included studies, extract the relevant data, and assess the quality of evidence utilizing the Grading of Recommendations Assessment, Development, and Evaluation system and the risk of bias using the Cochrane RevMan tool. The second reviewer will assess the quality of 25% of the included studies to assess interrater agreement. Any disagreement will be solved by a third reviewer. Only studies of high and moderate quality will be included for narrative synthesis.

**Conclusions:**

NHS policy assumes that increasing usage of personal health records by citizens will reduce demand on health care services. There is limited evidence, however, that the use of health apps can improve patient outcomes, and, to our knowledge, this is the first systematic literature review aiming to identify important design features of the personal health record which may improve medication adherence in the adult population with long-term conditions.

**Trial Registration:**

PROSPERO CRD42017060542; https://www.crd.york.ac.uk/prospero/display_record.php?RecordID=60542 (Archived by WebCite at http://www.webcitation.org/6zeuWXxVh)

**Registered Report Identifier:**

RR1-10.2196/9778

## Introduction

The annual National Health Service (NHS) England spend on medication was £15.5 billion in 2015 and the volume of medication prescribed in by NHS England rises every year [1-3]. The World Health Organization (WHO) reported that the average medication adherence in patients with long-term conditions in developed countries is approximately 50% [4]. According to the WHO, there is a need to acquire more data related to medication adherence from all age band subgroups [4]. It is estimated that, in the UK, more than a third of patients with at least one long-term illness do not adhere to their medication regime [5]. Medication nonadherence is associated with higher number of hospitalizations, adverse drug reactions, nursing home admissions, and an increase in health care and social costs [6].

A number of systems are currently employed to use information and communication technologies (ICT) to store, manage, and employ health and medical information. The use of ICT for NHS health care policy was made clear in the 2002 report, *Securing Our Future Health: Taking a Long-Term View* [7], even though patients’ electronic access to their health records had already been planned in *The NHS Plan 2000* [8]. Following that, the NHS developed the Summary Care Record and HealthSpace programs to explore the development and application of shared electronic health records (EHRs) and personal health records (PHRs) [9]. The NHS Future Forum highlights the importance of patient access to their online GP health records to assist in the development of a self-care and self-management culture [10].

NHS policy documents and frameworks such as the *Personalised Health and Care 2020* (P2020) report [11] and the *Five Year Forward View* [12] specify that the NHS needs to harness the power of technology. The aim is to enable patients to make correct choices and to support clinicians by providing access to all necessary data and assisting the clinicians to make the most of technology available and these data. The P2020 claims that unless the gap between care and technology closes, patients may experience “unnecessary levels of preventable ill health” [11]. It also provides evidence of the growing demand for technology in England, as evidenced by the fact that 59% of all UK citizens have a smartphone. It also compares the health care sector to other safety-critical industries and it argues that digital tools and technologies, such as mobile apps, improve self-management of patients’ health [11].

There are four main terms in general use for structured health care information systems, namely electronic medical records (EMRs), electronic patient records (EPRs), EHRs, and PHRs [13]. Although there have been attempts to differentiate the definitions of EMR, EPR, and EHR [14-16], in practice, these terms lack precision and are often used interchangeably [13]. We have adopted the term “EHR” in this protocol. The definition of a generic EHR is “a repository of information regarding the health status of a subject of care, in computer processable form” [13,17].

There are multiple definitions of a PHR. Generally, the term “PHR” emerged from “EHR” and can be defined as “health records related to patient care that are controlled by the patient” [18]. Although there are paper based PHRs, in this protocol we refer to PHRs that are electronic and accessible via mobile devices [18].

Based on the PHR definitions provided by Cruickshank [9], Paton [19], Rohers [18], and Archer [20], we identified the common denominators and the following PHR definition is used throughout this protocol and the systematic literature review:

PHRs are online systems that include collections of patients’ health care and medical data, which utilize health informatics standards to enable patients to share, organize, and manage these data according to their own views. [21]

**Figure 1:**
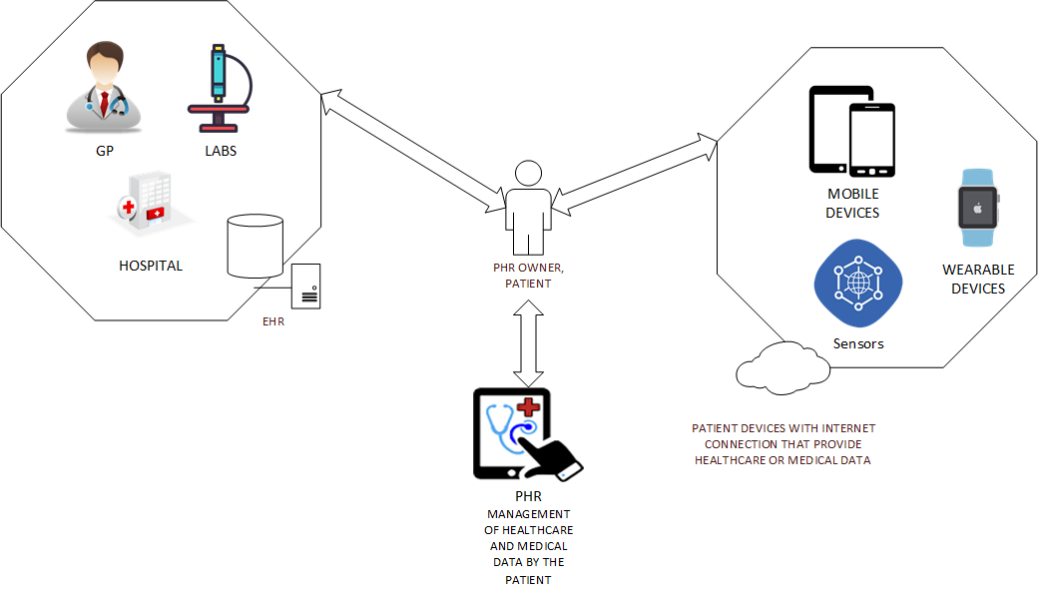
Description of the differences and concepts between electronic health records (EHRs) and personal health records (PHRs) [15,18]. GP: general practitioner.

This definition is agnostic to the type of PHR, which can be defined as tethered [16,20,22] or standalone [18,20]. A tethered (tied) PHR includes features that are not patient-controlled; thus, it can be connected to the data source, including the cloud and institutional EHRs [16,18,20]. Untethered or standalone PHRs’ main feature is that the patient-user is the only one permitted to enter, maintain, and self-manage data related to their own health conditions [20,23]. Based on the above definition, this review will also include studies that use copies of personal health data on storage devices, such as smart cards and USB sticks. Figure 1 illustrates how the EHR and PHR differ for health care and medical records.

PHRs are typically important for patients that are suffering from chronic conditions, whom gain the most value from the PHR and have a higher adoption rate for PHR use [20,24-26]. Some PHR characteristics, as derived by the literature [20,24-26], are summarized in Table 1.

The adoption of PHRs is global, for example, PHRs are expected to be adopted by 75% of patients in the USA by 2020 [27]. In Australia, a national tethered PHR system has been launched [28] and in Canada and Denmark there are health centers which offer PHRs to their patients [9]. Furthermore, the NHS England is working toward a greater adoption of PHRs [11,12,29]. There is a growing focus on the adoption of PHRs worldwide because, in addition to geographically targeting the major economies such as the USA and UK, studies have also been conducted in middle- and low-income countries to evaluate, improve, and quantify the benefits of PHR use in global health [9,30-35].

The scope of this review is global and there are no geographical restrictions on this study. The use of PHRs not only varies between different groups of patients, but it also varies among studies [36]. Patients that have a long-term condition or an illness that requires recurrent care are more likely to use a PHR than patients who claim to be in good health [37]. The adoption of PHRs worldwide started around 2003 [9], so all the evidence is recent, and this remains an immature and rapidly developing field. Illustrating this, early in 2011 Google decided to terminate their PHR product (Google Health) due to its low impact and adoption rate [9,38].

**Table 1:** Summary of personal health record (PHR) characteristics as derived by the literature [20,24-26].

PHR Characteristic	Examples
Administrative	Booking appointments, paying bills
Clinical features	View lab test results, view prescriptions, add medical history
Online access	May provide access to electronic health record data
Managed by patient	The patient’s data are controlled by patient
Data repository	Organizing health information, documenting symptoms, documenting medication dosages
Improves communications	Between patients and/or patient-doctor
Personalization	Provides individualized and tailored clinical information to patients
Medication adherence reminders	Might be alarms or text messages etc

NHS England engaged in a “landscape review” in 2015 to identify how local NHS organizations and commercial companies are using PHRs [39]. Most research and quality of life and care schemes that the NHS is currently referencing have been published after 2011 [11,36,40,41]. This context has informed our selection of a suitable date range for the literature searches.

There are many claimed benefits of PHRs, such as (1) the ability of PHRs to improve patient outcomes, (2) decrease in care costs, (3) to give patients the ability to self-manage their health, (4) an increase access to care especially in remote areas, (5) empowerment of patients, and (6) to improve medication adherence [18-20,24-26,36,42,43].

Medication adherence can be defined as “the extent to which a person’s behavior towards their medication intake, corresponds with agreed recommendations from a health care provider” [44]. Medication adherence is the preferred terminology, but some sources still use the word “compliance,” which many consider to be dated as it is a more restrictive term and less patient centered [45]. The ABC taxonomy [46] was selected as the conceptual framework for medication adherence in this study since it is well cited, it includes the time dimension, and it is considered more comprehensive than the WHO five interacting dimensions that affect adherence [44]. The ABC taxonomy states that there are three components to medication adherence: initiation (the time until the first dose has been taken), implementation (the extent to which a patient’s dosage consumption corresponds to the prescribed dose regimen) and discontinuation (stop taking the medication) [46]. Medication adherence and persistence are closely related and often persistence is incorporated in the notion of adherence [46,47]. Medication persistence can be defined as the extent to which patients adhere over time [45], in other words it is the time between the medication initiation until the medication discontinuation [46]. According to some authors, medication compliance and medication adherence are synonymous [46], with the latter not only to be introduced as a less aggressive term to describe the same phenomena but also to provide the patient with a sense of self-control and self-management of their treatment [48,49]. Concordance is another important term related to adherence to prescribed medication, which reflects the need to reach an agreement between a patient and the prescriber by which health beliefs are accounted for. A concordant consultation would be expected to lead to enhanced adherence to medication, as the prescribing process would involve the patient in the clinical decision making [48].

Some studies indicate that polypharmacy has a negative effect on medication adherence [44,50,51]. Polypharmacy is defined as the parallel use of multiple medications by one patient, for complex or multiple conditions [52-54]. Polypharmacy can signify “the prescribing of either many drugs (appropriately)” or “too many drugs (inappropriately)” [55]. Our focus extends to either use of polypharmacy, since the impact of PHRs on either polypharmacy or simple prescribing is unknown.

Medication adherence is a well-known challenge in health care [44,56-58], and is related to a large number of factors such as side effects [59], forgetfulness [57], or effective self-management and is affected by psychological factors and beliefs [60]. Although a number of strategies and interventions have been identified to assist patients’ medication adherence [58,61], they have had limited success.

NHS policy assumes that increasing the usage of health apps by citizens will reduce demand on health care services.

However, the quality of the literature about the use of health apps to improve patient outcomes is often questionable [62].

### Aim and Objectives

The aim of this systematic review is to identify important design features of the electronic PHR that may improve medication adherence in the adult population with long-term conditions.

#### Primary Objective

The primary objective of this systematic review is to identify the important design features of the electronic PHR which may improve medication adherence in the adult population with long-term conditions.

#### Secondary Objectives

- Identify the PHR design features that improve medication adherence in the cases of:
  - Polypharmacy;
  - Specific long-term condition groups;
- Identify if there is a correlation between participants’ demographic characteristics, their usage of PHRs, and their medication adherence;
- Explore how implementation factors affect the outcomes.

## Methods

This protocol complies with the requirement of the Preferred Reporting Items for Systematic Reviews and Meta-analyses Protocol (PRISMA-P 2015) including the PICOS elements (participants, interventions, comparators, outcomes and study design) highlighted in Table 2 [63].

**Table 2:** Summary of the PICOS elements (participants, interventions, comparators, outcomes and study design) included and excluded in the systematic review.

Variable	Inclusion criteria	Exclusion criteria
Participants	Humans Adults with at least one long-term condition Patients that can self-administer their medication Patients that are able to communicate freely and able to self-manage their medication Patients treated outside the hospital only	Animals Pregnant, cancer, or terminally ill patients Adults with medically serious problems that are not classified as long-term conditions Patients that require assistance with taking their medication Patients unable to communicate or unable to self-manage their medication. Inpatients or patients living in care homes
Intervention	Interventions of any type, intensity and frequency, that aim to investigate the effect of electronic PHRs^a^ in medication adherence, concordance, compliance or persistence.	N/A^b^
Comparators	N/A	N/A
Outcome	Any outcome related to the effect of electronic PHRs in medication adherence, concordance, compliance or persistence	N/A
Study design or type	Studies or literature reviews published in the last fifteen years, without any geographical restriction	Abstract-only reports without any references, commercial studies, party political statements, general discussion papers, magazine or newspaper articles, withdrawn abstracts or articles, protocols of reviews

^a^PHR: personal health record.

^b^N/A: not applicable.

### Search and Selection Strategy

High heterogeneity of the data is expected, in terms of target diseases, interventions, outcome measures, and study types. Therefore, a meta-analysis is avoided in favor of a qualitative analysis. A narrative synthesis [64] of the peer-reviewed medical and nursing literature as indexed in PubMed/MEDLINE, PubMed Central, Association for Computing Machinery digital library, Emerald Insight, Science Direct, BioMed Central, and Cumulative Index to Nursing and Allied Health Literature will be undertaken. The Cochrane Database of Systematic Reviews and the Cochrane Central Register of Controlled Trials will also be searched and any abstract-only reports without any citations will be excluded. Additional papers derived manually from the reference lists of the selected articles or studies, as well as from Research Gate and Google Scholar during the screening process, will also be included. Conference proceedings will also be searched using the Web of Science and IEEE Xplore databases. Discovery services from ProQuest will be used to include theses and dissertations on the search. All published studies, as described above, reported during the past fifteen years will be considered. Two reviewers (EA, PJS) will screen the included studies.

Papers in English will be considered for this review. The studies selection will be managed using Mendeley Desktop v 1.17.9 and Mendeley Web.

The search strategy was developed iteratively, based on trial searches, using the PICOS framework [63], together with a university librarian, and takes into consideration the methods section of previous systematic literature reviews in the field. The search strategy used is:

(phr OR “personal health record” OR “patient portal”) AND adult* AND (“chronic disease” OR “chronic illness” OR “chronic condition” OR “long term disease” OR “long term illness” OR “long term condition”) AND

“medication compliance” OR “medication adherence” OR “medication concordance” OR “medication persistence”

The search includes the following MeSH [65] terms: personal health records, medication adherence, and chronic disease.

The strategy does not include the terms “p.h.r.” nor “P.H.R.,” since these terms obscured the preliminary results by adding an unnecessary load of marketing and human resources related results, which are clearly out of the scope of this research. The search terms of the strategy were combined with Boolean Operators (ie, “AND” and “OR”). During preliminary searches, the word PHR was replaced by the words “medication record” or “medication profile” based on pharmacist advice; this search yielded very similar results in PubMed Central and, in fact, excluded 2 studies. A search that initially seemed promising was including the word “medication” and the word “adherence” by themselves (ie, “medication” AND “adherence”). A similar preliminary search excluded the word medication altogether. These two searches yielded tens of thousands of hits, which initially seemed promising, but, upon inspection, it was apparent that either the papers were investigating PHRs as recreational software, commonly for depression and weight loss, or the papers were investigating PHRs in conjunction with adherence to general therapeutically regimens such as weight loss and gym attendance.

### Participants

As illustrated in Table 1, the inclusion and exclusion criteria for the participants are the following. Studies that include adult patients with any long-term disease and use any type of self-administering medication will be assessed for inclusion. For this review, adult is defined as ≥18 years of age.

Studies that include adult patients with cancer, who are terminally ill, pregnant, or have any other problems which make patients unable to communicate freely and self-manage their medication will be excluded. Studies that include only inpatients or care home residents will be excluded.

### Intervention

Interventions researching influences which affect patient medication adherence will be included in this review as described in Table 1. Included studies will include interventions of any type, intensity, and frequency, which aim to investigate the effect of PHRs on medication adherence, compliance, persistence, and concordance.

The interventions may initially be grouped as:

- Interventions that explore the effect of PHR on medication adherence [44,46,66], compliance [45,46], persistence [66], and concordance [45];
- Interventions that explore how and how much patients use PHRs and the effect this use might have in medication adherence, compliance, persistence and perceptions;
- Interventions that explore the notion of polypharmacy in adult patients with multiple conditions and how PHRs and technology in general may be of assistance.

### Outcome

The primary outcome of the studies included in the review is medication adherence. However, medication adherence, compliance, persistence, and/or concordance are complicated terms to measure and often depend on the authors’ point of view, background, and the authors’ definitions of the above terms [48]. Furthermore, there is often a confusion surrounding the differences between the terms, for example, the difference between medication adherence and persistence [56]. Due to this complexity, an inclusive approach will be used to determine the outcomes of the included studies.

### Data Extraction

The data extraction forms were created based on the National Institute for Health and Care Excellence data extraction forms [67] and the data extraction chapter from the Cochrane Collaboration [68]. In cases of missing data on the PICOS elements, an email will be sent to the authors of the study. If there is no response within two weeks, a second email will be sent, and if there is still no response from the authors, the study will be excluded. The data extraction forms were designed to collect all the data needed to address the review questions and to follow the data synthesis strategy. The forms were piloted on a random selection of 10 of the included studies to assess any potential issues (Multimedia Appendix 1).

The following information will be extracted from each study:

1. Basic study characteristics (eg, title, authors, journal, abstract, keywords, publication, aim)
2. Study design and study period
3. Population characteristics (eg, age, number of participants, chronic illness etc)
4. Intervention characteristics (eg, length of use, design features, technological characteristics, vendor of PHR, type of PHR)
5. Outcome measures (eg, self-reported, clinical outcomes, medication adherence ideal)
6. Outcomes (eg, primary and secondary outcomes involving medication adherence, quality of life, and polypharmacy)

Besides the above data, additional information will be documented for the quality assessment and risk of bias analysis, as described below.

### Quality Assessment and Risk of Bias

Each eligible study will be assessed for validity and quality of evidence, using the Critical Appraisal Tools written by The Joanna Briggs Institute [69]. If there are studies that are eligible for inclusion, but have missing data, the authors of these studies will be contacted to see if these data can be obtained and used in this review.

Each eligible study will also be assessed for risk of bias using the Cochrane handbook 2011 [70]. The Grading of Recommendations Assessment, Development, and Evaluation (GRADE) tool [71] will be used to assess the quality of the aggregate evidence for each outcome, based upon five factors: risk of bias, inconsistency, indirectness, imprecision, and publication bias. The quality of evidence will be rated as high, moderate, low, or very low for every outcome.

The main reviewer (EA) will evaluate all the included studies, the second reviewer (PJS) will evaluate 25% of the included studies and interrater reliability will be calculated [72]. Any disagreement will be solved by consulting a third reviewer. Only studies of high and moderate quality, as defined by GRADE [73], will be included in the review.

### Data Synthesis and Analysis

Based on the aim of this review, the wide range coverage of research designs, multiple interventions and outcomes, as well as the expectation of high heterogeneity, all the data that will be extracted from the studies will be analyzed narratively using an interpretative framework [64,74]. To ensure that the narrative analysis will be of good quality, the “Guidance on the Conduct of Narrative Synthesis in Systematic Reviews” [64] will be followed, which is in line with the Cochrane data synthesis and analysis guidelines [74].

The narrative analysis will attempt an investigation into the similarities and the differences between the outcomes of different studies and an exploration of themes (patterns) in the data. The guidelines [64,74], include the following four stages of a narrative synthesis in reviews:

1. Development of a theory of how the intervention works, why, and for whom. The initial theory and the familiarization with the data will be achieved based on the development of a textual description of the studies, which will be produced systematically including, where possible, the same information for all studies and in the same order [64].
2. Development of a preliminary synthesis of the findings of the included studies. The preliminary synthesis will be developed using a tabular analysis of the studies in multiple tables, using Microsoft Excel, followed by a thematic analysis, which will systematically identify the recurrent themes across the included studies [64].
3. Exploration of the relationships in the data between and within studies. A conceptual model will be developed to explore relationships in the data, which will group similar findings and identify relationships between these groups, providing visualization of the possible relationships across studies [64].
4. Assessment of the robustness of the synthesis. A Best Evidence Synthesis (BES) approach will be followed BES is typically applied during the selection process and is primarily concerned with the methodological quality of the included studies. BES guidelines require that all the included studies will meet the minimum standards for relevance and quality of evidence, and that all the extracted data will be systematically extracted based on the data extraction forms. Therefore, the decision regarding the “strength of evidence” will be made early in the review process [64].

The analysis will be conducted in an iterative and abductive way and the results will be thoroughly discussed with the other two authors (PJS and HH).

### Potential Amendments

There is no intention to amend the protocol; thus, the possibility of outcome reporting bias will be reduced. However, if any amendments are needed during the review process, they will be clearly and comprehensively reported.

## Results

There is no requirement for ethical review since this study is secondary research. The final report of the systematic review in the form of a scientific paper will be published in a peer-reviewed journal. Findings may further be presented at conferences and be submitted to relevant NHS authorities. We also plan to include an updated version of this systematic review in the author’s thesis.

## Discussion

This research is limited to include only articles that have outcomes related to the effect of electronic PHRs in medication adherence, concordance, compliance, or persistence, rather than also including other outcomes such as quality of life. In this sense, the review will focus exclusively on articles that measure medication adherence as a primary or secondary outcome. This review is limited to obtain articles published either in ICT or health care and medical portals. This means that a lot of ICT related literature is unobtainable, since it in not included in the academic literature, but is commercial or governmental work. The aim of this review is to identify the essential design features of the PHR that assist adults with at least one long-term condition to adhere with their medication, without taking into consideration adolescents nor adults that are not considered chronically ill or terminally ill, have cancer, or are pregnant.

## Appendix

Multimedia Appendix 1 Data extraction forms.

## Abbreviations

EHR: electronic health record
EMR: electronic medical record
EPR: electronic patient record
GRADE: Grading of Recommendations Assessment, Development, and Evaluation
ICT: information and communication technologies
P2020: Personalised Health and Care 2020
PHR: personal health record
PICOS: participants, interventions, comparators, outcomes, and study design
PRISMA: Preferred Reporting Items for Systematic Reviews and Meta-Analyses
WHO: World Health Organization
