# Supplementary material for: Important Design Features of Personal Health Records to Improve Medication Adherence for Patients with Long-Term Conditions: Protocol for a Systematic Literature Review
Source: JMIR Res Protoc. 2018 Jun 28;7(6):e159. doi: 10.2196/resprot.9778 (PMC6116916; doi:10.2196/resprot.9778)
Supplement: Multimedia Appendix 1 [file resprot_v7i6e159_app1.pdf]

APPENDIX A – DATA EXTRACTION FORMS

Publication Characteristics

Study Index	**Authors**	**Title**	**Publication**	**Abstract**	**Keywords**	**Aim**
**1**						
2						

Location and Time data

Study Index	**Duration of study**	**Year of study**	**Location of Study**
**1**			
2			

Polypharmacy

Study Index	**Number of medications**	**Comorbidities**	**Number of Comorbidities**
**1**			
2			

Study Characteristics

Study Index	**Study type**	**Chronic Disease**	**Participants**	**Interventions**	**Control Group**	**Primary Outcomes**	**Secondary Outcomes**	**Type of PHR**	**PHR Vendor**	**Data collection method**	**Data analysis method**	**Adherence assessment**	**Adherence Ideal**	**Ethical Considerations**	**Notes**
**1**															
2															
